# The Evaluation of Health Care Services for Children and Adolescents With Post–COVID-19 Condition: Protocol for a Prospective Longitudinal Study

**DOI:** 10.2196/41010

**Published:** 2023-04-11

**Authors:** Chiara Rathgeb, Maja Pawellek, Uta Behrends, Martin Alberer, Michael Kabesch, Stephan Gerling, Susanne Brandstetter, Christian Apfelbacher

**Affiliations:** 1 University Children’s Hospital Regensburg Hospital St. Hedwig of the Order of St. John University of Regensburg Regensburg Germany; 2 Member of the Research and Development Campus Regensburg (WECARE) Hospital St. Hedwig of the Order of St. John Regensburg Germany; 3 Children’s Hospital Technical University Munich Munich Germany; 4 Institute of Social Medicine and Health Systems Research Otto von Guericke University Magdeburg Magdeburg Germany

**Keywords:** post-COVID, long COVID, post–COVID-19 condition, PCC, post–COVID-19 syndrome, PCS, CFS/ME, children and adolescents, health care services, EQ-5D, SDQ, PROMIS, DSQ-PEM, COVID-19, pediatrics, child, adolescent, service delivery, healthcare delivery, healthcare service, care network, healthcare, therapeutic service, healthcare utilization, patient reported outcome

## Abstract

**Background:**

Some children and adolescents suffer from late effects of a SARS-CoV-2 infection despite a frequently mild course of the disease. Nevertheless, extensive care for post–COVID-19 condition, also known as post–COVID-19 syndrome, in children and young people is not yet available. A comprehensive care network, Post-COVID Kids Bavaria (PoCo), for children and adolescents with post–COVID-19 condition has been set up as a model project in Bavaria, Germany.

**Objective:**

The aim of this study is to evaluate the health care services provided within this network structure of care for children and adolescents with post–COVID-19 condition in a pre-post study design.

**Methods:**

We have already recruited 117 children and adolescents aged up to 17 years with post–COVID-19 condition who were diagnosed and treated in 16 participating outpatient clinics. Health care use, treatment satisfaction, patient-reported outcomes related to health-related quality of life (the primary endpoint), fatigue, postexertional malaise, and mental health are being assessed at different time points (at baseline and after 4 weeks, 3 months, and 6 months) using routine data, interviews, and self-report questionnaires.

**Results:**

The study recruitment process ran from April 2022 until December 2022. Interim analyses will be carried out. A full analysis of the data will be conducted after follow-up assessment is completed, and the results will be published.

**Conclusions:**

The results will contribute to the evaluation of therapeutic services provided for post–COVID-19 condition in children and adolescents, and avenues for optimizing care may be identified.

**International Registered Report Identifier (IRRID):**

DERR1-10.2196/41010

## Introduction

### Background

COVID-19, caused by the novel coronavirus SARS-CoV-2, is considered a multi-organ disease with varying symptom patterns [1]. Frequently occurring characteristics are fever, cough, headache, myalgia, or fatigue and in some cases, intestinal symptoms [2,3]. Research has focused on the treatment of acute COVID-19 cases, which mainly occur among elderly patients and people with preexisting comorbidities like diabetes or cardiovascular disease [4-6]. These persons belong to a risk group among whom a more severe form of the infection can also occur [7].

Children and adolescents are less affected by severe forms of the infection and usually have a mild course of disease [8,9]. Nonetheless, some of them still suffer from long-term consequences of the infection, referred to as “long COVID,” post–COVID-19 syndrome (PCS) or post–COVID-19 condition (PCC) [10], which not only affect risk groups, but also people with a milder form of COVID-19 or even asymptomatic SARS-CoV-2 infection [11-13]. Those persistent clinical symptoms vary considerably but can be very restricting for the individual [10].

Currently, there is no clear and uniform definition of PCC [14]. According to the National Institute for Health and Care Excellence, 3 different phases of an infection with SARS-CoV-2 should be distinguished [10]. An acute COVID-19 infection is classified by signs and symptoms that can last up to 4 weeks. The next phase is between 4 and 12 weeks and is termed ongoing symptomatic COVID-19. If signs and symptoms last longer than 12 weeks, are consistent with SARS-CoV-2 infection, and cannot be explained by any other diagnosis, this is called PCC. The commonly used term “long COVID” includes ongoing symptomatic COVID-19 and PCC [10]. According to the World Health Organization (WHO), the following definition is used [15]:

Post COVID-19 condition occurs in individuals with a history of probable or confirmed SARS CoV-2 infection, usually 3 months from the onset of COVID-19 with symptoms that last for at least 2 months and cannot be explained by an alternative diagnosis.... Symptoms may be new onset following initial recovery from an acute COVID-19 episode or persist from the initial illness. Symptoms may also fluctuate or relapse over time. A separate definition may be applicable for children.

Most recently, a modified Delphi consensus was used to suggest a first research definition of PCC in children and adolescents that complements the clinical case definition in adults proposed by the WHO [16]:

Post-COVID-19 condition occurs in young people with a history of confirmed SARS-CoV-2 infection, with at least one persisting physical symptom for a minimum duration of 12 weeks after initial testing that cannot be explained by an alternative diagnosis. The symptoms have an impact on everyday functioning, may continue or develop after COVID infection, and may fluctuate or relapse over time. The positive COVID-19 test referred to in this definition can be a lateral flow antigen test, a PCR test or an antibody test.

Frequently reported long-term symptoms in children and adolescents after COVID-19 are fatigue, headache, sleep disturbance, concentration difficulties, abdominal pain, muscle and joint pain, cough, chest pain, loss of appetite or weight, disturbed smell or anosmia, and rash [11,12]. This symptom pattern appears similar to the one seen in adults [11,17]. Another symptom of PCC in children and adolescents is Kawasaki-like hyperinflammation, also known as multisystem inflammatory syndrome in children associated with COVID-2019 (MIS-C) or pediatric inflammatory multisystem syndrome temporally associated with SARS-CoV-2 (PIMS-TS) [11,18]. Patients with this condition have persistent fever and high levels of inflammation in which several organs are affected [19,20]. In addition, acute SARS-CoV-2 infection can cause myalgic encephalomyelitis/chronic fatigue syndrome (ME/CFS) [21,22]. The main symptoms of this complex, severe disease are fatigue and exercise intolerance with symptoms worsening after minimal exertion, which is referred to as postexertional malaise (PEM) [23,24]. In general, data on PCC in children and adolescents is still scarce [25,26].

The prevalence of PCC has been estimated at 0.8% to 13% in pediatric studies with uninfected controls [12,27,28]. The Follow CoKiBa study in Regensburg, Germany, found mild, long-lasting symptoms in 10% of children and adolescents aged up to 14 years who had a previous SARS-CoV-2 infection and severe, long-lasting symptoms in at least 3% of those who were affected with a mild, acute form of COVID-19 [29].

Due to the complexity of its symptoms, the diagnosis and treatment of PCC is time-consuming and often difficult [10]. In addition, children and adolescents can be affected by pandemic-related mental disorders [30,31], and differential diagnosis for these disorders and PCC is difficult [32]. Therapy should be carried out depending on a comprehensive differential diagnosis and the patient’s symptoms. However, no data or recommendations on how to provide treatment are yet available [1,33].

There is an urgent need to create a comprehensive care network for children and adolescents with PCC. Models and structures of care are needed that enable interdisciplinary, somatic, and psychological diagnostics with subsequent comprehensive therapy. In Bavaria, Germany, such a network of physicians and specialized outpatient clinics, named Post-COVID Kids Bavaria (PoCo), has been established to provide specific care for young patients with PCC, including a specialized Post-COVID Fatigue Centre (PCFC). This should ensure that those who are affected receive appropriate care as close to home and with as little stress as possible. To the best of our knowledge, no other studies have been published that focus on the evaluation of health care services for children and adolescents with PCC.

### Aim of the Study and Hypotheses

The overarching aims of this study are to describe care pathways in the context of PCC in children and adolescents and to evaluate the effects of health care services from the perspective of affected children and adolescents within and outside the PoCo network.

We hypothesize that the health care being provided within the PoCo network is improving the subjective health status of young patients with PCC. Table 1 describes the specific objectives and hypotheses of the study.

**Table 1:** Objectives and hypotheses of the study.

Objectives	Hypotheses
Documentation of care pathways, including care structures used and therapeutic measures prescribed, with regard to post–COVID-19 condition.	Patients will have many contacts with the health care system within and outside the Post-COVID Kids Bavaria (PoCo) network.
Evaluation of the effects of therapeutic programs.	The patients’ health status and quality of life will improve over time.
Evaluation of satisfaction with different care structures and therapeutic programs.	The patients will be mostly satisfied with care for post–COVID-19 condition and will use the different care structures.

## Methods

### Study Design

This study is taking place within the PoCo network, which follows long-term conditions after COVID-19 in children and adolescents. The aim of the PoCo network is to provide optimal care for children and adolescents affected by PCC. This involves pediatricians and general practitioners (GPs) in private practice, as well as 16 specialized pediatric outpatient clinics all over Bavaria, including one with focus on the treatment of ME/CFS (PCFC). Thus, the network comprises ambulatory and stationary health care institutions throughout Bavaria. Statewide provision of care is ensured by trained, practicing physicians and supplemented by telemedical services.

Patients with severe forms of PCC are cared for in special outpatient clinics. If post-COVID ME/CFS is suspected, patients are treated in or together with the PCFC in Munich. Patients with severe limitations, such as pain, sleep disorders, cognitive problems, or orthostatic intolerance are cared for as inpatients in the pediatric rehabilitation center in Berchtesgaden or in the pediatric pain clinic in Garmisch-Partenkirchen. The latter has expertise in treating children with ME/CFS.

**Figure 1:**
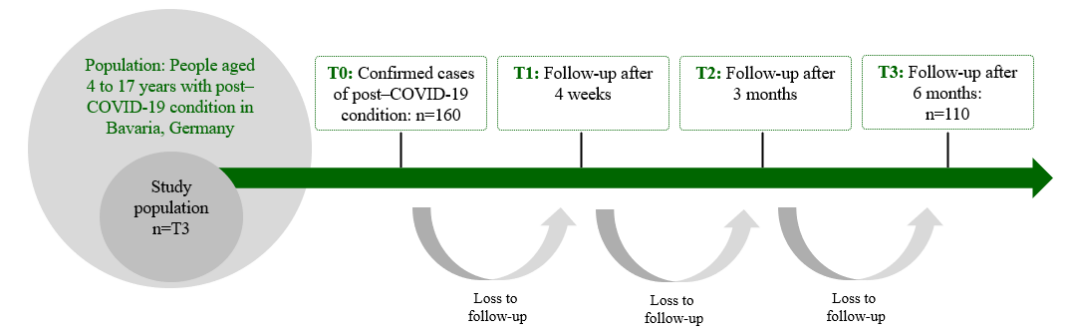
Planned study sample and measurement points.

This study is dedicated to health-services research within the PoCo network. It has a longitudinal design (Figure 1). Patients were included in the study after the (suspected) diagnosis of PCC by participating special outpatient clinics. If patients had been treated before, a retrospective assessment of diagnoses and treatments was performed. Patients are assessed at baseline (ie, inclusion in the study; T0), after 4 weeks (T1), after 3 months (T2), and after 6 months (T3) using routine data, interviews, and questionnaires. The primary outcome is subjective health status after 6 months, measured by the visual analogue scale (VAS) of the EQ-5D [34].

### Sample and Recruitment

#### Study Sites

Pediatricians or GPs in private practice in the PoCo network all over Bavaria recruited patients and referred them to the participating outpatient clinics, where they were included in the study. Patients could also be included directly via the special outpatient clinic without having been referred by a pediatrician or GP.

#### Patients

Male and female patients aged 4 to 17 years were included in this study if they had a positive test for SARS-CoV-2 and showed clinically evident PCC symptoms or had a COVID-19 infection in the family environment in combination with PCC-typical symptoms. Textbox 1 shows the inclusion and exclusion criteria.

**Inclusion and exclusion criteria.**

**Inclusion criteria**

- Children and adolescents, male and female, aged 4 to 17 years
- Proficient German language skills
- Evident clinical abnormalities or symptoms of post–COVID-19 condition with one or both of the following (at least 4 weeks earlier):
  A positive test for SARS-CoV-2, including any or all of the following: a polymerase chain reaction test, antigen test, and SARS-COV-2 antibodies
  A verified SARS-CoV-2 infection in the family environment
- Current treatment at one of the participating outpatient clinics
- Written consent of the parents and the patient (depending on age)

**Exclusion criteria**

- Age <4 years or >17 years
- Nonproficient German language skills
- No positive test for SARS-CoV-2 or confirmed infection in the patient’s family
- No current treatment at one of the outpatient clinics
- No written consent

### Sample Size and Patient Flow

Based on the primary outcome (subjective health status, measured with the VAS), a sample size calculation was performed. Under the assumption of an effect size *d*=0.35, a significance level α=.05, and power β=.80, the calculated sample size was n=55. However, we assumed that there would be some patients who declined participation in the study, even after inclusion, or dropped out. Therefore, we planned to include 80 patients who were treated within the PoCo network. Patients included and cared for in the PCFC will be evaluated separately. The same assumptions for sample size and dropout were made for these patients, resulting in the inclusion of an additional 80 patients. Thus, the result of the sample size calculation was n=160.

### Measurements

Table 2 provides an overview of the variables that are measured for answering the research questions.

A detailed list of all variables is presented in Multimedia Appendix 1 [10,16,34-38].

**Table 2:** Overview of variables, constructs and scores, and measurement instruments.

Domains and variables/constructs			Scores, instruments, and data sources		Time point	
**Sociodemographic characteristics**						T0
	Patient’s age, gender, current education/health care facility and health insurance status, family household size, other children living in the household, place of residence, parents’ migration status, level of education and vocational education	Proxy questionnaire, hospital information system				
**General medical characteristics**						T0
	Height, weight, current medication, chronic conditions and diseases	Hospital information system				
**Medical characteristics relating to COVID-19 and its treatment**						T0
	History of SARS-CoV-2 infection, symptom patterns, use of ambulatory and stationary health care, comorbidities, treatment and medication of comorbidities, COVID-19 vaccination status	Hospital information system				
**Characteristics of health care use**						T1, T2, T3
	Visits to physicians and therapists, recommended and used treatment, usefulness of treatment, recommended and used medication or supplements, visits to special outpatient clinic, length of stay and frequency of visits at special outpatient clinic, use of telemedicine, inpatient admission, days of absence from school or work due to illness, counselling on school attendance	Proxy questionnaire via telephone interview, hospital information system				
**Patient-reported experience measures**						T1, T2, T3
	Treatment satisfaction	Proxy questionnaire via telephone interview				
**Patient-reported outcome measures**						T0, T1, T2, T3
	Health-related quality of life	EQ-5D-Y [34], visual analogue scale [34]				
	Fatigue	Patient-Reported Outcomes Measurement Information System (PROMIS) Pediatric Short Form version 2.0–Fatigue 10a [36]				
	Postexertional malaise	DePaul Symptom Questionnaire Focusing on Post-exertional Malaise (DSQ-PEM) [37]				
	Mental health	Strength and Difficulties Questionnaire (SDQ) [38], proxy questionnaire (age ≥12 years: self-reported questionnaire)				

#### Sociodemographic Characteristics

Sociodemographic information about the patients and their families characterizing the sample was obtained with a paper-based questionnaire (described in the Data Sources and Data Collection sections) handed out at baseline. These variables included patients’ age, gender, level of education and health insurance, family household size, other children living in the household, place of residence, migration status, and parents’ level of education and vocational education.

#### General Medical Characteristics

General medical information was also asked for at baseline, to obtain an overview of the patient’s general state of health. Patients’ height, weight, current medication used, and chronic conditions and diseases were assessed with routine data and questionnaires.

#### Medical Characteristics Relating to COVID-19 and Its Treatment

Different parameters relating to COVID-19 were recorded at baseline: history of SARS-CoV-2 infection, symptom patterns, use of ambulatory and stationary health care, comorbidities, treatment and medication against comorbidities, and COVID-19 vaccination. The information was obtained from routine data and questionnaires.

#### Characteristics of Health Care Use

To evaluate use of health care services within and outside the PoCo network, the following variables are recorded at T1, T2, and T3 by means of a telephone interview with the parents: visits to physicians and therapists, recommended and used treatment, usefulness of treatment, recommended and used medication or supplements, days of absence from school or work due to illness, visits to a special outpatient clinic, length of stay and frequency of visits at the special outpatient clinic, use of telemedicine, inpatient admission, and counselling on school attendance. Telemedicine includes structured correspondence of pediatricians and GPs with specialists via the post-COVID–specific PädExpert PraxisApp before admission to a hospital, as well as follow-up visits by phone, email, or video conference.

#### Patient-Reported Experience Measures

Information on patient experience is captured by the variable “treatment satisfaction.” Data is collected via telephone interviews at the measurement timepoints T1, T2, and T3.

#### Patient-Reported Outcome Measures

The primary outcome is the VAS, scored on a scale from 0 to 100, of the EQ-5D-Y. Remaining questions of the EQ-5D-Y are answered on a 3-point Likert scale [34]. Secondary outcomes are fatigue and PEM. Fatigue is assessed with the PROMIS Pediatric Short Form version 2.0–Fatigue 10a questionnaire. The questionnaire comprises 10 items, which are answered on a 5-point Likert scale [36]. A total score is calculated by building the sum of the individual item scores, with higher scores indicating greater fatigue [39]. The DePaul Symptom Questionnaire Focusing on PEM (DSQ-PEM) provides information on the presence of PEM by asking about the frequency and severity of different symptoms [37]. Further, the emotional problems scale of the Strength and Difficulties Questionnaire (SDQ) is used to examine mental health. Items are answered on a 3-point Likert scale. By summing up the individual item scores, a total score is determined that indicates whether a patient shows abnormalities for mental problems, with higher scores implying higher risk for abnormalities [38]. Patient-reported outcome measures (PROMs) are assessed at all measurement points with proxy questionnaires for children aged 4 to 11 years and self-report questionnaires for adolescents aged 12 years and older. Only adolescents aged 12 years and older provide information about fatigue.

### Data Sources and Data Collection

Data are derived from various sources. Characteristics relating to COVID-19 and its treatment, as well as general medical characteristics, were obtained from the hospital information system by health care providers and entered into an electronic database (DB), Qnome (Maganamed). Sociodemographic characteristics were assessed via paper questionnaires. These questionnaires were already filled out at baseline by the parents of the patients. Characteristics of health care use are measured via telephone interviews at different time points (Table 2). These questions are also answered by the parents. Some sociodemographic characteristics and characteristics of health care use were assessed via routine data from the hospital information system. PROMs are obtained via paper questionnaires at all time points from the participants themselves or from their parents if they are younger than 12 years. All questionnaires are sent to the study participants by mail. Completed paper questionnaires are manually entered into the electronic DB using the individual IDs of the participants.

### Data Management and Quality Assurance

Qnome is used for electronic data collection and data management. The study participants were assigned to the Qnome DB after their parents (and, depending on age, the participants themselves) had confirmed their written consent to participate in the study. Within this overarching DB, however, separate DBs were set up for the individual data sources and types, which are stored separately from each other. A distinction is made between the DB of the trust center (TC) for personal data, the clinical DB for clinical data (including routine data) and the health-services research DB for health-services research data. The data of the study participants were pseudonymized and provided with different IDs: for the clinical data, the clinical data ID (C-ID) and for the health-services research data, the health-services research ID (H-ID). Each participant was assigned a randomly generated pseudonymized C-ID and H-ID. The C-ID was communicated to the attending physicians, who performed a structured clinical examination as part of patient care and documented and store all findings in the clinical DB with this C-ID. The H-ID is only used by the health services research team for their data. The TC is responsible for the provision of the different IDs and the linking to the personal data.

**Figure 2:**
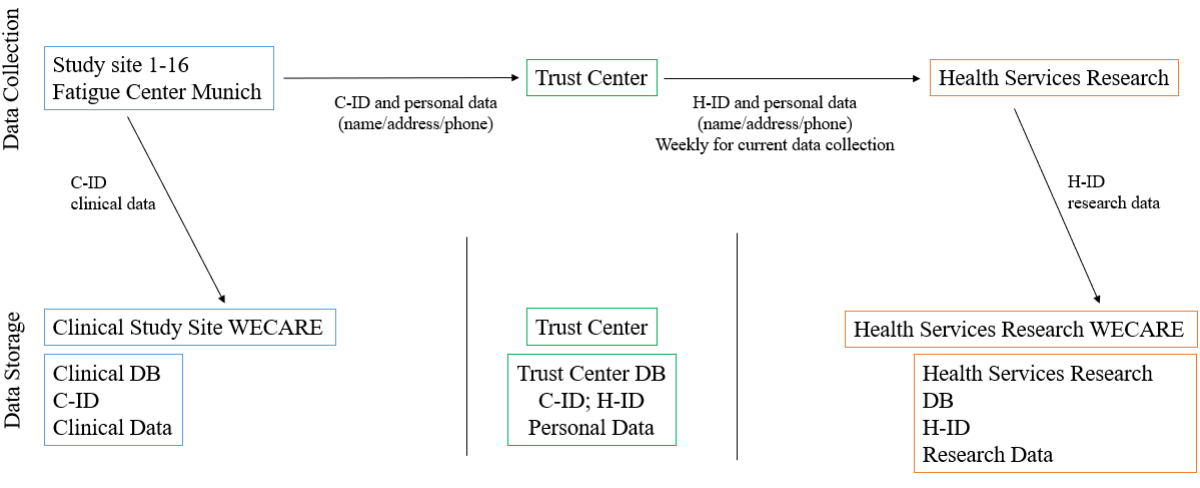
Data collection and data storage. C-ID: clinical data ID; H-ID: health services research ID; DB: database; WECARE: Research and Development Campus Regensburg.

For the data collection for the health-services research (Figure 2), contact data are forwarded by the TC with the corresponding H-ID to the health-services research team. For this purpose, a list of persons to be contacted is compiled weekly and transmitted to the health services research team. The transmission of the personal data for contacting is carried out via Citrix ShareFile (Citrix), an internet-based platform for data transmission that is detached from the study DBs. The data are stored on the local server of the Barmherzige Brüder Hospital Regensburg for a short period of time. At the designated follow-up time points, the health-services research team contacts the person with custody to conduct the interview or to fill in the questionnaire. By means of the H-ID, the answers to the interview conducted by the health-services research team can be entered into the health-services research DB. After successful contact, the weekly list with the contact data and IDs will be destroyed.

**Figure 3:**
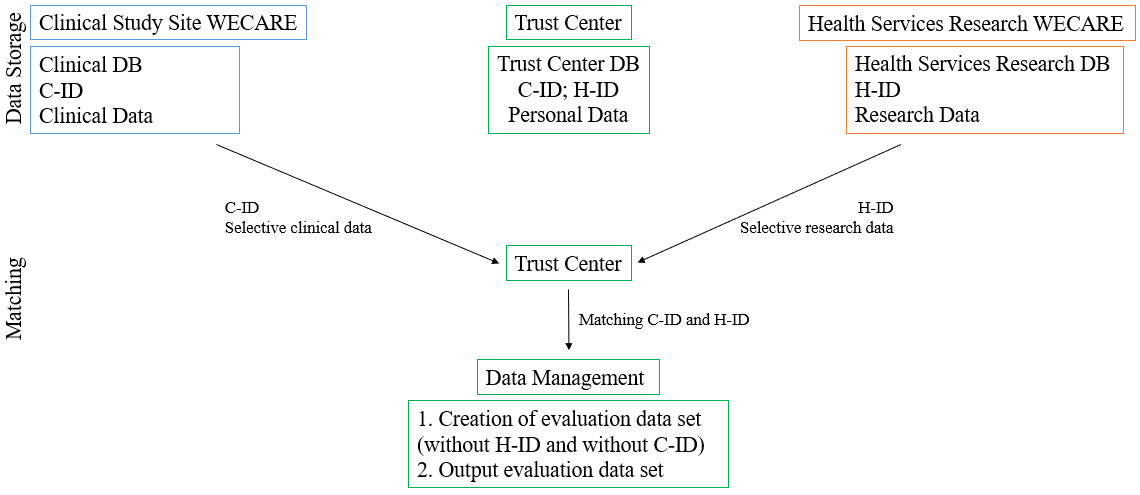
Data storage and matching (only for joint evaluation, if necessary). C-ID: clinical data ID; H-ID: health services research ID; DB: database; WECARE: Research and Development Campus Regensburg.

When data assessment is complete, data sets will be prepared that do not contain the names or contact details of the study participants (Figure 3).

### Statistical Analysis

Plausibility checks of the data are performed continuously during data collection and before data analysis. Patients who drop out during the course of the study will be compared to patients who stay in the study by comparing their general characteristics, such as age, education, and migration background, at baseline. When information is missing in the questionnaire, queries are sent to participants. Descriptive statistics will be used to analyze patient characteristics and to describe care pathways, diagnoses, and treatments. Parents’ education will be transformed to categories according to the Comparative Analysis of Social Mobility in Industrial Nations (CASMIN) classification, and the children’s BMI will be calculated using their height and weight [40]. Additionally, PROMs will be analyzed for each measurement point using descriptive statistics.

Therapeutic programs will be analyzed using a pre-post study design. The Wilcoxon signed-rank test for matched samples will be used, as outcome data (eg, VAS) are not assumed to be normally distributed. VAS scores at baseline and after 6 months will be compared in order to detect significant improvement after therapeutic interventions. A difference of 10 points on the scale indicates a clinically relevant change. Analyses of the minimal important difference (MID) for PCC in children and adolescents are not available. This assumption of 10 points was derived pragmatically. Half an SD is taken as the MID for quality-of-life measures [41]. A study on acutely ill children, chronically ill children, and children from the general population found a mean VAS score of 77.1 (SD 21.3) [42]. The value 10 is slightly less than half of 21.3. Studies in older people show lower SDs (eg, 18.5) [43]. Therefore, a minimal clinically important difference of 10 seems to be a pragmatic determination. All analyses will be carried out using SPSS (version 28; IBM Corp) and R (version 4.1.1; R Foundation for Statistical Computing).

### Ethics and Consent

The study received original approval on November 29, 2021, and approval of an amendment on March 10, 2022, from the ethics committee of the University of Regensburg (21-2691-101). The study received original approval on April 7, 2022, and approval of an amendment on April 26, 2022, from the ethics committee of the medical faculty of the Technical University Munich (2022-103 S-SR). All data collected will be pseudonymized. Written informed consent was obtained from all study participants at baseline, from their parents, and, depending on their age, from the participants themselves. Participation in the study is voluntary, and participants were reassured that they are free to withdraw from the study at any time without consequences.

## Results

The recruitment process for the study ran from April 2022 to December 2022. Unfortunately, only 117 children and youth were included in the study. The planned sample size could not be reached within the period scheduled for recruitment. Interim analyses will be carried out and a full analysis of the data will be conducted after follow-up assessment is completed. The results will be published.

## Discussion

The PoCo network aims to improve the care of young patients with PCC by providing comprehensive, multimodal, and intersectoral health services in Bavaria. The main focus is to ensure that comprehensive differential diagnosis is made and that subsequent patient-specific therapy is provided whenever indicated. The established network facilitates access to health services. Using a longitudinal study design, we will be able to evaluate the health care services provided within the PoCo network using outcomes assessed from the patient’s perspective. To this end, we use extensively evaluated and widely used tools [37,44-46]. It is assumed that health-related quality of life, fatigue, PEM, and mental health will improve over time in children and adolescents participating in this study.

In addition, this study will improve our knowledge about medical characteristics relating to COVID-19 and its treatment as well as health services and treatments used by young patients with PCC. Risk groups can be identified that are particularly affected by the long-term effects of SARS-CoV-2, including patients with ME/CFS. The prospective evaluation of health care services for children and adolescents with PCC brings some challenges, including consideration of the heterogeneous symptoms of PCC and the complexity of therapeutic measures. In addition, the different age groups make it difficult to identify outcome measures suitable for all participants, and severely affected patients may have problems completing the self-reported instruments due to fatigue or exertion intolerance. Moreover, as in many longitudinal studies, patients might drop out of the study early on, due, for example, to the presumed improvement of symptoms through the care provided in the network. We acknowledge that the study is a single-group study and lacks a control group. We will therefore not be able to study the effects of the health services provided in the PoCo network in comparison to usual care or the improvement in health status caused by natural recovery over time. Also, we will not be able to investigate the effects of the different treatment strategies used in the PoCo network in a comparative manner. Nevertheless, this study will contribute to the evaluation of the health care services provided in the PoCo network in response to the needs of those children and adolescents who suffer from prolonged sequelae after COVID-19.

## Appendix

Multimedia Appendix 1 Detailed overview of variables.

## Abbreviations

C-ID: clinical data ID
CASMIN: Comparative Analysis of Social Mobility in Industrial Nations
DB: database
DSQ-PEM: DePaul Symptom Questionnaire focusing on post-exertional malaise
GP: general practitioner
H-ID: health-services research ID
ME/CFS: Myalgic encephalomyelitis/chronic fatigue syndrome
MID: minimal important difference
MIS-C: Multisystem Inflammatory Syndrome in Children Associated with Coronavirus Disease 2019
PCC: post–COVID-19 condition
PCFC: Post-COVID Fatigue Centre
PCS: post–COVID-19 syndrome
PEM: postexertional malaise
PIMS-TS: pediatric inflammatory multisystem syndrome temporally associated with SARS-CoV-2
PoCo: Post-COVID Kids Bavaria
PROM: patient-reported outcome measure
PROMIS: Patient-Reported Outcomes Measurement Information System
SDQ: Strength and Difficulties Questionnaire
TC: trust center
VAS: visual analogue scale
WHO: World Health Organization
