# Correction: The Evaluation of Health Care Services for Children and Adolescents With Post–COVID-19 Condition: Protocol for a Prospective Longitudinal Study

**DOI:** 10.2196/48338

**Published:** 2023-04-25

**Authors:** Chiara Rathgeb, Maja Pawellek, Uta Behrends, Martin Alberer, Michael Kabesch, Stephan Gerling, Susanne Brandstetter, Christian Apfelbacher

**Affiliations:** 1 University Children’s Hospital Regensburg Hospital St. Hedwig of the Order of St. John University of Regensburg Regensburg Germany; 2 Member of the Research and Development Campus Regensburg (WECARE) Hospital St. Hedwig of the Order of St. John Regensburg Germany; 3 Children’s Hospital Technical University Munich Munich Germany; 4 Institute of Social Medicine and Health Systems Research Otto von Guericke University Magdeburg Magdeburg Germany

In “The Evaluation of Health Care Services for Children and Adolescents With Post–COVID-19 Condition: Protocol for a Prospective Longitudinal Study” (JMIR Res Protoc 2023;12:e41010) the authors made 5 reference corrections.

In Table 2, column 2, row 13, the reference in the following text:

Patient-Reported Outcomes Measurement Information System (PROMIS) Pediatric Short Form version 2.0–Fatigue 10a [35]

Has been changed to:

Patient-Reported Outcomes Measurement Information System (PROMIS) Pediatric Short Form version 2.0–Fatigue 10a [36]

In Table 2, column 2, row 14, the reference in the following text:

DePaul Symptom Questionnaire Focusing on Post-exertional Malaise (DSQ-PEM) [36]

Has been changed to:

DePaul Symptom Questionnaire Focusing on Post-exertional Malaise (DSQ-PEM) [37]

In Table 2, column 2, row 15, the reference in the following text:

Strength and Difficulties Questionnaire (SDQ) [37], proxy questionnaire (age ≥12 years: self-reported questionnaire)

Has been changed to:

Strength and Difficulties Questionnaire (SDQ) [38], proxy questionnaire age ≥12 years: self-reported questionnaire

The references in following sentences under Patient-Reported Outcome Measures:

The questionnaire comprises 10 items, which are answered on a 5-point Likert scale [35]. A total score is calculated by building the sum of the individual item scores, with higher scores indicating greater fatigue [39]. The DePaul Symptom Questionnaire Focusing on PEM (DSQ-PEM) provides information on the presence of PEM by asking about the frequency and severity of different symptoms [36]. Further, the emotional problems scale of the Strength and Difficulties Questionnaire (SDQ) is used to examine mental health. Items are answered on a 3-point Likert scale. By summing up the individual item scores, a total score is determined that indicates whether a patient shows abnormalities for mental problems, with higher scores implying higher risk for abnormalities [37].

Have been changed to:

The questionnaire comprises 10 items, which are answered on a 5-point Likert scale [36]. A total score is calculated by building the sum of the individual item scores, with higher scores indicating greater fatigue [39]. The DePaul Symptom Questionnaire Focusing on PEM (DSQ-PEM) provides information on the presence of PEM by asking about the frequency and severity of different symptoms [37]. Further, the emotional problems scale of the Strength and Difficulties Questionnaire (SDQ) is used to examine mental health. Items are answered on a 3-point Likert scale. By summing up the individual item scores, a total score is determined that indicates whether a patient shows abnormalities for mental problems, with higher scores implying higher risk for abnormalities [38].

In the “Discussion” section, the references in the phrase:

To this end, we use extensively evaluated and widely used tools [36,44-46].

Have been changed to:

To this end, we use extensively evaluated and widely used tools [37,44-46].

The corrections will appear in the online version of the paper on the JMIR Publications website on April 25, 2023, together with the publication of this correction notice. Because this was made after submission to PubMed, PubMed Central, and other full-text repositories, the corrected article has also been resubmitted to those repositories.

