# Supplementary material for: The Evaluation of Health Care Services for Children and Adolescents With Post–COVID-19 Condition: Protocol for a Prospective Longitudinal Study
Source: JMIR Res Protoc. 2023 Apr 11;12:e41010. doi: 10.2196/41010 (PMC10132031; doi:10.2196/41010)
Supplement: Multimedia Appendix 1 [file resprot_v12i1e41010_app1.pdf]

Table 1. Detailed overview of variables

**Characteristics**	**Variables**	**Units or Categorical Response Options**
Socio-demographic characteristics	Age	Years (4-17)
	Gender	Male, female
	Current education/care facility	Day care center / nursery school, primary school, middle school, Realschule, Gymnasium, special school, vocational school / apprenticeship, technical school (FOS) / vocational high school (BOS), University
	Health insurance of patient	Public health insurance, state aid (Beihilfe), private health insurance, overseas health insurance, other claims to health insurance, no health insurance
	Family household size	Number of persons living in the household
	Further children living in the household	Number of children living in the household
	Place of residence	Postal code
	Parents´ migration status (at least one parent born outside of Germany)[35]	Yes, no; if yes: country of origin, years living in Germany since immigration
	Parents´ level of school education	Currently attending school, no graduation, completion of compulsory basic secondary schooling, certificate of secondary education, advanced technical leaving certificate, general advanced leaving certificate, school leaving examination in second-chance education, other
	Parents´ level of vocational education	Currently in training, no professional qualification, completed apprenticeship, completed training at technical school, Bachelor, Master/state examination, PhD, other
General medical characteristics	Height, weight	cm, kg
	Current medication	Yes, no; If yes: name of medication
	Chronic conditions/diseases	Yes, no; If yes: type of chronic disease
Medical characteristics relating to COVID-19 and its treatment	History of SARS-CoV-2 infection [10,16]	Number of infections<br>Time after infection (weeks)
	Symptom patterns	Smelling disorder, taste disorder, shortness of breath, respiratory distress, bronchial hypersensitivity, decreased resilience, dizziness, chest pain, fever episodes, joint paints, muscle pain, hair loss, stomach ache, vomiting, diarrhea,

		conspicuous stool admixtures (blood or mucus), weight loss, headache, insomnia, profound pathological fatigue, concentration disorders, significant drop in academic performance, depression, anxiety
	Use of ambulatory and stationary health care	No use<br>Yes: Outpatient, stationary
	Comorbidities	Yes, no; If yes: type of comorbidity<br>(Comorbidities in the fields of neuropediatric, cardiology, pneumology, gastroenterology, neuropsychology)
	Treatment and medication of comorbidities	Treatment: Physiotherapy, exercise therapy, occupational therapy, psychotherapy, speech therapy, homeopathy, acupuncture, traditional chinese medicine (TCM), others.<br><br>Medication: yes, no; If yes: name of medication
	COVID-19 vaccination	yes, no; If yes: number and type of vaccination (Biontech, Moderna, AstraZeneca, Johnson&Johnson, other)
Characteristics of health care use	Visits to physicians and therapists (before special outpatient clinic)	Number of visits: pediatrician, general practitioner, internist, (pediatric) cardiologist, (pediatric) pulmonologist, ear-nose-throat doctor, (pediatric) neurologist, (pediatric) psychiatrist, dermatologist
	Recommended and utilised treatment (before, through and additional to special outpatient clinic)	Before special outpatient clinic: exercise/sport, physiotherapy/manual therapy/physical therapy, respiratory therapy, occupational therapy, speech therapy, psychotherapy, services provided by a non-medical practitioner (e.g. aromatherapy, homeopathy, TCM, kinesiology, bioenergetics, etc.), service with the osteopath, service of the chiropractor, services provided by a curative teacher, other services<br><br>Through special outpatient clinic/ Additional to special outpatient clinic: exercise/sport, physiotherapy/manual therapy/physical therapy, respiratory therapy, pain therapy, smell and taste therapy, hyperbaric oxygen therapy, occupational therapy, speech therapy, psychotherapy, TCM, acupuncture, relaxation techniques, pacing, other therapy
	Usefulness of treatment	5-point Likert scale
	Recommended and utilised medication or supplements (before,	Medication: yes, no; If yes: name of medication<br>Supplements: yes, no; If yes: name of supplement

	through and additional to special outpatient clinic)	
	Visits to special outpatient clinic	Name of outpatient clinic
	Length of stay and frequency of visits at special outpatient clinic	Number of visits<br>Length of stay: <30min, 30min - <1h, 1 - <2h, 2 - <3h, 3 - <4h, 4 - <5h, 5 - <6h, >6h
	Use of telemedicine	Contacted professional: Pediatrician, psychiatrist, psychologist, social pedagogue/social pedagogue, teacher<br>Number of contacts<br>Average Length (hours)<br>Usefulness (5-point Likert scale)<br>Reason for usage: General, time saving, less effort, answering questions<br>Use of PädExpert (App): Yes, No
	Inpatient admission	Yes, no; if yes: length (days); current inpatient admission, inpatient admission completed, inpatient admission cancelled<br>Usefulness of inpatient admission (5-point Likert scale)
	Days of absence from school/work due to illness	Number of absent days
	Counselling on school attendance	Yes, no: counselling on general school topics, avatar, compensation for disadvantages<br>Usefulness (5-point Likert scale)
PREM	Treatment satisfaction	5-point Likert scale
PROMs	Health related quality of life	EQ-5D-Youth [34], VAS [34]
	Fatigue	PROMIS® Pediatric Short Form v2.0 – Fatigue 10a [36]
	PEM	DSQ-PEM [37]
	Mental Health	SDQ [38]
